# Editorial: Global population aging – Health care, social and economic consequences, volume II

**DOI:** 10.3389/fpubh.2023.1184950

**Published:** 2023-04-17

**Authors:** Mihajlo Jakovljevic, Narimasa Kumagai, Seiritsu Ogura

**Affiliations:** ^1^Institute of Advanced Manufacturing Technologies, Peter the Great St. Petersburg Polytechnic University, Saint Petersburg, Russia; ^2^Institute of Comparative Economic Studies, Hosei University, Tokyo, Japan; ^3^Department of Global Health Economics and Policy, University of Kragujevac, Kragujevac, Serbia; ^4^Faculty of Economics, Seinan Gakuin University, Fukuoka, Japan; ^5^Faculty of Economics, Hosei University, Tokyo, Japan

**Keywords:** population, global, economics, costs, health, social, demographics, policy

While there had been some isolated periods of fertility decline in Europe in the last two centuries, the so-called third Demographic Transition phenomenon became broadly recognized mainly over the past three decades. It consists of falling female fertility, improved early childhood survival, extended longevity, and ultimately the rapid growth in the proportion of the older adults population in a society (1). Among the major underlying causes, the following are listed: growing living standards, improvements in medical services, public health, public education, the sexual revolution and consequent absorption of unpaid female labor into the labor markets. In the process, the families undergo significant changes in their social role; large or multi-generation families dissolve into nuclear families, keeping the reproduction, consumption, education, and health production functions but losing most of the functions for production and intergenerational resource transmission (2).

The transition to an aging society brings severe financial challenges to all institutions in any economy. Even most developed countries that had foreseen these problems coming for two decades are still struggling to find money to pay for the bulging retirement income and healthcare costs for the growing older adults population. Their transition has been made more difficult by the new revolutionary medical technologies, extremely costly, that are targeted to save the life of a relatively small number of patients. To what extent should public money be spent to save the life of the few, mostly the older adults?

Another unsolved health policy problem for high-income countries is long-term care for the older adults. With the longevity and differential gender mortalities, we observe a swelling population of the very old, overwhelmingly female and poor, living alone. Only two or three decades ago, they would have been taken care of by their children or their families, but with family ties weakening, the government is now asked to provide necessary social services for them. How should we finance these costs? How can we preserve incentives for family caregiving? (3).

These problems are no less serious, if not more, for most middle-income countries. Compared with the historical experiences of developed countries, their demographic transition is taking place much faster. While, at the moment, they may be enjoying huge “demographic dividends,” they are bound to face much larger bills for retirement income, healthcare costs, and long-term care costs for their disproportionately large older adults populations (4).

There are significant gaps in the literature, particularly for less recognized middle-income regions, magnitudes of the demographic shocks involved, the size of the expected costs of aging and the distribution of these costs over generations. Although only some developed countries have succeeded in preparing for their demographic shocks, how are these countries preparing for the upcoming aging shocks? (5).

Today we have evidence that aging is occurring even among some of the poorest countries, bringing double burdens to their national health system. Still coping with the responsibility of infectious diseases, injuries, and high neonatal mortality, these communities face a high toll of chronic non-communicable diseases usually associated with old age in more developed countries (6).

The most comprehensive set of studies on basic medical insurance, private health insurance, healthcare, evaluation of financial sustainability of basic pension, evaluation of medication usage, aging, fertility and other topics are provided by authors from all around the globe.

With ~8,500 employees, Semmelweis University is a leading institution in medicine and health sciences in Hungary and the Central European region. The Center of Preventive Services' mission is to provide comprehensive preventive services, such as health promotion programs, health status assessments, lifestyle counseling, and medical risk assessment. Monitoring and defining the effectiveness of a workplace health promotion/disease prevention program is a must, but evaluation methods developed for and applied in health promotion in various (primarily occupational) settings have frequently fallen short of the ideal.

Researchers from the Medical University of Sofia, Bulgaria, conducted an intriguing study regarding the identification of potentially inappropriate medication and potential prescribing omissions in older patients with cardiovascular diseases in Bulgaria. Krustev et al. carried out a questionnaire-based survey among 543 senior patients. This study's findings show a high percentage of potentially inappropriate medication among older patients with polypharmacy. With Bulgaria's aging population, the economic burden of polypharmacy, and the prevalence of cardiovascular diseases, it is critical to address potentially inappropriate medication use in cardiovascular patients.

Aging is a significant trend in the changing age structure of the world's population, and it is a major economic and social issue impeding the development of all nations. The data for this study, conducted by Zhang et al., came from the China Health and Retirement Longitudinal Survey (CHARLS) in 2013, 2015, and 2018. The findings show that health shocks hampered the improvement of the shared prosperity of the middle-aged and older adults in rural areas, with daily activities having the most significant negative impact. Yet, the government is expected to broaden the scope and proportion of essential medical insurance payments, gradually close the medical insurance gap between urban and rural residents, and achieve basic public service equalization.

Wang C. et al. conducted an exciting study on a similar topic, examining whether intergenerational support was associated with depressive symptoms differently among urban and rural Chinese older participants. A total of 3,498 participants were included from nine pairs of urban subdistricts and rural villages. The 10-item Center for Epidemiological Studies Depression Scale was used to assess depressive symptoms, and a self-designed questionnaire was used to evaluate intergenerational support mechanisms (financial, instrumental, and emotional). The findings support modernization theories that propose weakened economic functions but strengthened emotional ties in higher-developed societies.

A curious study by Zhu et al. focused on predicting models and associated factors on the fertility behaviors of the floating population in China. The data for this study came from the 2016 China Migrants Dynamic Survey, which used a stratified three-stage random sample proportional to the population and collected information *via* anonymous questionnaires. This study provided a total of 168,993 valid questionnaires. The factors influencing the reproductive behavior of the floating population were complex, including social health services, family income, and the burden of urban living. The high-accuracy prediction model of the population's childbearing behavior could assist relevant departments in better predicting and intervening in the development of the floating population and improving their fertility rate, ultimately alleviating population aging and promoting economic growth.

Chen et al. conducted a study of the private health insurance's (PHI's) complementary role in health using the three-wave balanced panel data of the China Health and Retirement Longitudinal Survey (CHARLS), which is conducive to providing evidence for PHI's policy expansion and encouraging the public to participate in PHI, which is insufficient in China in comparison to social health insurance (SHI). Participating in supplementary PHI can significantly improve the insured's health status and significantly impact patients with chronic diseases. PHI development should be encouraged further, while health disparities between income groups should be addressed.

Using the STOP-Bang questionnaire combined with the Epworth Sleepiness Scale (ESS) in screening for obstructive sleep apnea (OSA) in the population, Zheng et al. have done good work on screening patients for OSA. The combination of STOP-Bang (≥3) and ESS significantly improved its specificity for predicting OSA. This screening method can help doctors conduct stratified management based on patients' OSA risk levels, identifying high-risk patients and ultimately reducing the harm caused by OSA.

Wang Y. et al. from the Liaoning University of China investigated how to alleviate the demographic structure dividend in the face of an aging population, increasing domestic demand and accelerating domestic movement. In this popular topic, there are numerous theoretical assumptions and potential solutions to this problem. To comprehensively stimulate consumption growth, it is necessary to accelerate urbanization development progress and rely on the Internet. According to the estimation results, the urbanization level, Internet usage rate, and developmental level of the tertiary industry played a significant positive promotion role in the consumption structure upgrade.

A researcher from the Jiangxi University of Finance and Economics, China, provided exciting insight regarding population aging as a global issue. Chen examined the characteristics of the aging population of insureds participating in basic pension insurance before randomly simulating the basic pension insurance's long-term financial situation in this analysis. The study discovered that the aging of insureds has a “fierce coming and slow decline” and “long-term seriousness.” He then examined the impact of the main parameter direction and degree on the fund's financial situation and the result of parameter value paths on the fund's final financial status to improve the fund's ability to strengthen its reserve. Among the many conclusions was the idea of going back to see if there was a way to enhance the basic pension insurance fund reserves.

In China, low fertility has become a major social issue. In this retrospective study conducted by Ning et al., data from the 2017 China General Social Survey have been collected. The results were exciting. Women's fertility intentions differ significantly *p* < 0.01 depending on their media use preferences, education level, and family income. New media use negatively influences women's fertility intentions, whereas traditional media use does not significantly influence women's fertility intentions. This paper argues that strengthening social trust and online agenda-setting is important for improving women's fertility intentions and that strategic information communication can change their perceptions of social trust. The government can utilize the new media agenda-setting to decrease work-family imbalance, thereby increasing fertility intentions. For women having lower identification with Chinese traditional gender roles, providing information that health losses of women with multiple roles can be reduced if the spouse shares the housework by engaging in frequent cleaning of the house (7) may be useful.

This Research Topic was created to address the challenges of global population aging, issues with basic and private health insurance, medication usage evaluation, and fertility issues, among other significant topics. The editors hope that these substantial and diverse subject contributions will expand existing knowledge and provide potential solutions to global problems. A diverse group of authors from academia, the pharmaceutical, medical device, and economics industries collaborated to present a comprehensive review of advanced understanding of various popular topics. This collection of articles is intended to pique the interest of other researchers and the general public.

## Author contributions

MJ has prepared the draft manuscript while MJ, NK, and SO have revised it for important intellectual content. All authors contributed to the article and approved the submitted version.
